# Fabrication and In Vitro Characterization of Novel Hydroxyapatite Scaffolds 3D Printed Using Polyvinyl Alcohol as a Thermoplastic Binder

**DOI:** 10.3390/ijms232314870

**Published:** 2022-11-28

**Authors:** Andrej Thurzo, Paulína Gálfiová, Zuzana Varchulová Nováková, Štefan Polák, Ivan Varga, Martin Strunga, Renáta Urban, Jana Surovková, Ľuboš Leško, Zora Hajdúchová, Jozef Feranc, Marian Janek, Ľuboš Danišovič

**Affiliations:** 1 Department of Orthodontics, Regenerative and Aesthetic Dentistry, Faculty of Medicine, Comenius University, 81250 Bratislava, Slovakia; 2 Institute of Histology and Embryology, Faculty of Medicine, Comenius University, 81104 Bratislava, Slovakia; 3 Institute of Medical Biology, Genetics and Clinical Genetic, Faculty of Medicine, Comenius University, 81108 Bratislava, Slovakia; 4 National Institute of Rheumatic Diseases, 92112 Piešťany, Slovakia; 5 Department of Inorganic Materials, Faculty of Chemical and Food Technology, Slovak University of Technology, 81237 Bratislava, Slovakia; 6 Department of Plastics, Rubber and Fibres, Faculty of Chemical and Food Technology, Slovak University of Technology, 81237 Bratislava, Slovakia; 7 Department of Physical and Theoretical Chemistry, Faculty of Natural Sciences, Comenius University, 84215 Bratislava, Slovakia

**Keywords:** regenerative dentistry, 3D printing, biomimetic, bioinspired materials, MSC, cell colonization, tissue engineering, regenerative medicine, oral bone, tissue regeneration, biocolonization, CDHA, MTT, LDH, SEM, FDM

## Abstract

This paper presents a proof-of-concept study on the biocolonization of 3D-printed hydroxyapatite scaffolds with mesenchymal stem cells (MSCs). Three-dimensional (3D) printed biomimetic bone structure made of calcium deficient hydroxyapatite (CDHA) intended as a future bone graft was made from newly developed composite material for FDM printing. The biopolymer polyvinyl alcohol serves in this material as a thermoplastic binder for 3D molding of the printed object with a passive function and is completely removed during sintering. The study presents the material, the process of fused deposition modeling (FDM) of CDHA scaffolds, and its post-processing at three temperatures (1200, 1300, and 1400 °C), as well it evaluates the cytotoxicity and biocompatibility of scaffolds with MTT and LDH release assays after 14 days. The study also includes a morphological evaluation of cellular colonization with scanning electron microscopy (SEM) in two different filament orientations (rectilinear and gyroid). The results of the MTT assay showed that the tested material was not toxic, and cells were preserved in both orientations, with most cells present on the material fired at 1300 °C. Results of the LDH release assay showed a slight increase in LDH leakage from all samples. Visual evaluation of SEM confirmed the ideal post-processing temperature of the 3D-printed FDM framework for samples fired at 1300 °C and 1400 °C, with a porosity of 0.3 mm between filaments. In conclusion, the presented fabrication and colonization of CDHA scaffolds have great potential to be used in the tissue engineering of bones.

## 1. Introduction

Hydroxyapatite (HA) has been used in regenerative medicine as an inert scaffold since the 1950s. Due to its high osteoconductivity and biocompatibility, it is widely used in bone tissue engineering [1,2]. Regenerative dentistry plays a promising role in all areas of dentistry, especially in periodontology and implantology, in the treatment of bone defects in teeth and implants [3].

Various craniofacial and dental surgical procedures require the reconstruction of bone defects caused by trauma, disease, tumor resection, or simply premature tooth loss. The dental arch, after tooth extraction, begins to lose bone in the corresponding part of the alveolar bone [4,5]. This effect may prevent the usual therapeutic scenario of dental implant placement due to the lack of supporting bone for the planned dental implant.

Globally, millions of bone graft procedures are being performed by clinicians annually to treat the rising prevalence of bone defects. Calcium phosphate-based biomaterials have excellent properties and are widely used to repair bone defects due to their similarity to the inorganic components of human bone. Hydroxyapatite (HA), as the thermodynamically most stable crystalline phase of CaP in aqueous solutions, is mostly used in the form of ceramics or composite frameworks with polymers [6]. It is considered to be a promising scaffold material also for dental and orthopedic implants due to its ideal biocompatibility and high osteoconductivity. The implant morphology modification has been extensively studied to regulate the host immune environment and further promote bone regeneration [7,8].

Current advances in HA-based biocomposites for bone tissue regeneration in regenerative dentistry are opening new therapeutical opportunities. Bone tissue is a nanocomposite constituted of an organic and inorganic matrix in which the collagen component and the mineral phase are organized in complex and porous structures. HA is, therefore, the most used ceramic biomaterial, as it mimics the mineral composition of human bone. However, this biomimetic material has poor mechanical properties, such as low tensile and compressive strength, making it less than ideal for bone tissue engineering. HA biocomposites are generally biocompatible, as demonstrated by most in vitro and in vivo studies in animal models [9].

**Figure 1:**
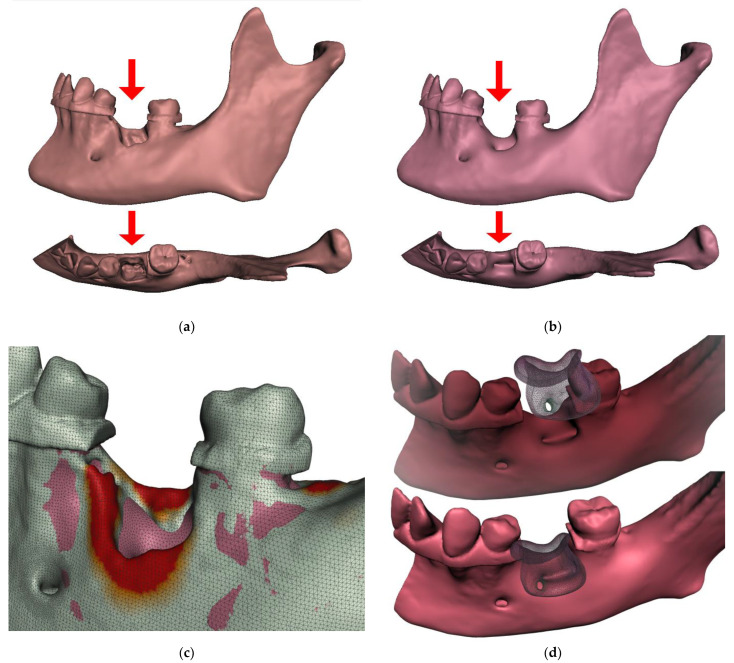
Following tooth extraction, the alveolar ridge undergoes an inevitable remodeling process. The concept of prospective clinical application of colonized 3D printed personalized scaffolds: (**a**) Extraction of a molar in the mandible can be the cause of an extensive loss of alveolar bone; (**b**) smoothing of the natural bone ridges and remodeling, typically leading to further recession of the alveolar bone level in this area; (**c**) a differential heat map of the comparison of aligned segmented CBCT models may reveal the extent of bone remodeling that has occurred; (**d**) a prospective scenario for the use of colonized scaffolds in clinical situations where an augmentation of alveolar bone is required.

The repair of critical size in alveolar bone defects is still an unmet clinical need, and in recent decades, materials scientists have made efforts to find effective technological solutions based on the use of suitable scaffolds. Although calcium phosphates are widely accepted as biomaterials for the fabrication of regenerative bone scaffolds, their processing into 3D devices with suitable cell-instructive properties is still hindered by insurmountable drawbacks [10]. Successful bone reconstruction requires the development and use of bone grafts that have structural, functional, and biological properties like those of natural tissue [11,12]. Research in this paper reports the feasibility of prospective clinical scenarios for bone augmentations personalized in 3D shape and on the cellular level. Such homologous bone scaffolds, individualized in shape and colonized with the patient’s own cells, are a tantalizing scenario for which effective and safe manufacturing still needs to be found. An approximation of the possible scenario is shown in Figure 1. As exemplified in Figure 1a, extraction of the mandibular first molar may result in extensive loss of alveolar bone, followed by smoothing of the natural bone ridges and remodeling, typically leading to further recession of the alveolar bone level in this area (Figure 1b). Cone beam computed tomography (CBCT) segmentation and matching of the two models can reveal the extent of bone remodeling (Figure 1c). Figure 1d shows a prospective scenario for the use of colonized scaffolds in clinical situations where an augmentation of the alveolar bone is required. The advantage of such an application is the ideal complementarity of the 3D shape and the biological compatibility.

Three-dimensional (3D) printing is a promising technology for a variety of healthcare applications, from regenerative medicine and tissue engineering to the production of clinical appliances [13,14,15,16,17]. From the comparison of conventional biofabrication methods with 3D printing, it is evident that the potential of 3D printing to produce on-demand, personalized, and complex products is unparalleled. Recent trends in the use of 4D materials (printed materials that change over time or in response to stimuli) can be used to overcome many of the inherent limitations of traditional 3D printing technologies [18]. Modern dental care will be dominated by two new trends: artificial Intelligence and regenerative dentistry [19,20,21,22].

Although the clinical application of bone printing technology is still in its infancy, the production of whole and functional bone parts characterized by an appropriate shape is an attractive and significant challenge in tissue engineering. Bone printing, such as cell source selection and achieving viable vascularization within the newly printed bone, is a major challenge in efforts to create 3D dense tissue, as this requires the development of an appropriate vascular supply. Biologically printed bone has been successfully implanted in preclinical models, and 3D-printed plastic, ceramic, or metal implants for bone tissue replacement [23] have been successfully transplanted into humans [24,25].

More than a decade ago, advances in polymer manufacturing enabled the development of hybrid systems consisting of multiple polymer formulations in different physical configurations. In 2009, Quigley et al. presented a hybrid polymer platform consisting of biodegradable polymer fibers. Taking advantage of the properties of conductive and biodegradable polymers, their scaffold was designed to promote directional axonal growth and migration of Schwann cells via the microstructure of the biodegradable polymer fibers [26]. In addition, a recent study by Wiatrak et al. 2021, showed that nanocrystalline apatite doped and co-doped with Li^+^ and Eu^3+^ ions might be a very attractive biomaterial for the regeneration of nervous tissue, where europium ions influence neuronal features even more strongly than doping with lithium alone [27].

The biopolymer presented in this study is polyvinyl alcohol (PVA), which is only used as a passive thermoplastic binder for 3D molding during 3D object printing. It is a water-soluble synthetic polymer that, when heated to temperatures above 250 degrees in the air, the thermal degradation oxidation processes are present, resulting in the formation of water and carbon dioxide as main products. Some of the most common medical applications of PVA are soft contact lenses, eye drops, embolization particles, tissue adhesion barriers, artificial cartilage, and meniscus. PVA has been explored in the production of fibers that align and promote proliferation and cell-cell interactions of renal cells [28] or has been explored in its oxidized form as a new polymer to produce nerve conduits [29]. PVA-based composite hydrogels are also promising materials with various biomedical applications. However, their mechanical and tribological properties often need to be modified. For example, the study of Feng et al. (2022) on the preparation of PVA gellan gum hydrogels showed the effects on their rheological and tribological properties [30].

The application of biomimetic strategies and bioinspired materials is widely used in the field of regenerative dentistry [31]. 3D printing of bone scaffolds can be performed using various 3D printing techniques such as FDM, powder sintering, and many others [24]. This is also true for pore geometry, which has a great influence on the cellular response. This is why 3D printing is such an attractive technology for bone tissue engineering, as it allows complete control and shaping of porosity. Calcium phosphate materials synthesized from natural sources have recently attracted some interest because they closely resemble natural bone and have better bioactivity than synthetic compounds [32].

Only recently has research been published highlighting the importance of surface properties. The study by Devi et al. (2022) designed a fucoidan from Sargassum ilicifolium incorporated in an osteoinductive scaffold comprising calcium crosslinked sodium alginate-nano hydroxyapatite-nano graphene oxide (Alg-HA-GO-F), which tends to serve as a bone graft substitute. The SEM revealed highly suitable surface properties, such as porosity and nanoscale roughness. The physical, structural, and enriching osteogenic potential results of Alg-HA-GO-F indicate that it can be a potential bone graft substitute for orthopedic applications [33].

The internal geometry of the scaffolds is only one aspect. In regenerative dentistry, the key aspect related to advanced dental implants, bone and soft tissue regeneration using autologous grafts or xenografts, allografts, their integration, and acceptance does not depend only on the material. The host response also plays a very important role through its vascularization [34].

As was highlighted, the chemical composition and surface topology of tissue-engineered scaffolds are two crucial parameters for regulating cell behavior [35], albeit processes following the colonization of the scaffold are also relevant to the cell differentiation aspect. For example, mechanical stimuli play a role in osteogenesis and chondrogenesis [36].

The use of mesenchymal stem cells (MSCs) in regenerative therapeutic procedures is becoming an increasingly important topic in medicine. Since the first isolation of MSCs derived from dental tissue, the properties and potential of these cells in regenerative dentistry have been intensively studied. Their multi-differentiation potential, self-renewal ability, and ease of access give them a key role in stem cell-based therapy. To date, several types of dental stem cells have been discovered, and their potential use can be found in most of the major branches of dentistry. Dental tissue-derived MSCs have been shown to be a valuable stem cell source with great therapeutic potential. Despite a respectable number of in vitro and in vivo studies on MSCs treatment in regenerative dentistry, there are still factors that need to be overcome in order to establish more predictable and reliable clinical protocols [37]. Dental stem cells are often used for oral regenerative applications [38]. Gingival stem cells are a limitless reservoir for regenerative medicine. Gingival tissue can be easily collected and represents an accessible source for the isolation of gingival mesenchymal stem cells (GMSCs). GMSCs are a subpopulation of gingival-derived mesenchymal stem cells that exhibit the characteristics of mesenchymal stem cells (MSCs), such as differentiation capacity and immunomodulatory properties. Dental stem cells are also expandable in vitro, genomically stable, and have the ability to maintain their stem cell properties over time. GMSCs should be considered as a good stem cell source for potential applications in tissue engineering and regenerative dentistry [39].

3D bioprinting in dentistry is unfortunately not yet close to clinical reality. Therefore, further research on the fabrication of ideal bio-inks with implantation into larger animal models in the oral environment is urgently needed for clinical implementation [40].

The current clinical reality is well reviewed in the recent study focused on available in vivo studies of oral bone tissue regeneration using MSCs. In general, unseeded scaffolds have shown limited regenerative potential. The bone regenerative potential of scaffolds enriched with MSCs can be influenced and improved by the addition of biomolecules, such as bone morphogenetic proteins, or the modulation of biomaterial features, such as pore dimension. Most of the studies used composite scaffolds or biomaterials with surface modifications, together with MSCs [41].

The aim of this paper is to introduce a composite filament based on hydroxyapatite material suitable for FDM 3D printing for novel scaffold fabrication with the perspective of full cellular and 3D shape personalization. This Paper also presents in vitro characterization of this material after colonization with MSCs.

## 2. Results

The scaffolds were printed in rectilinear/gyroid layer arrangement, and three sintering temperatures of 1200 °C, 1300 °C, and 1400 °C were used. The cells adhered well to both layer arrangements and were comparable to the control group. Most of the cells were observed on material fired at 1300 °C and 1400 °C, the fewest at the lowest temperature. None of the samples showed an increased rate of leaching of LDH into the solution. Scaffold material prepared at 1200 °C had a slight inhibitory effect on cell proliferation.

### 2.1. MTT Assay

**Figure 2:**
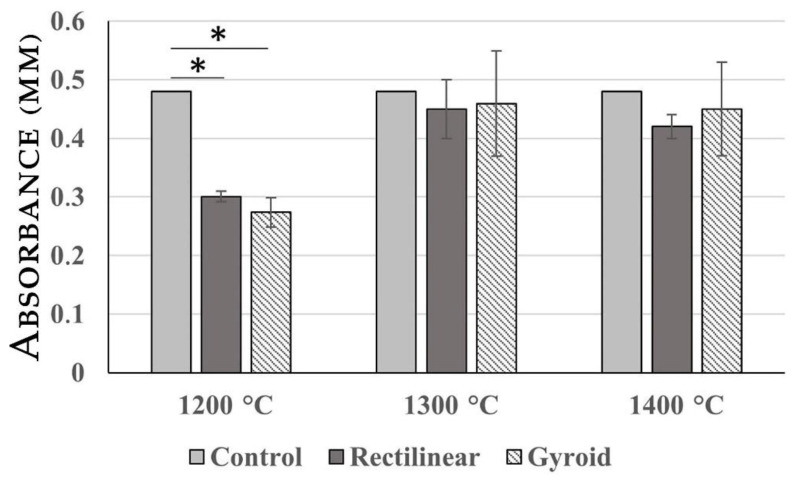
Results of MTT assay. The analyzed scaffolds processed at 1300 °C and 1400 °C were non-toxic. Only in the case of scaffolds processed at 1200 °C did we record a significant inhibitory effect when compared with the control. Asterisk (*) represents Extreme outlier which is an observation that lies an abnormal distance from other values.

A cytotoxicity study was conducted by MTT assay as a first step to evaluate the potential of fabricated materials. The results of the MTT assay presented in Figure 2 showed that all analyzed materials were non-toxic and the structure of the scaffold did not have a significant impact on the proliferation of MSCs. When compared with negative control, the best results were obtained in the case of material processed at 1300 °C and 1400 °C. However, the material prepared at 1200 °C had an inhibitory effect on cell proliferation.

### 2.2. LDH Release Assay

**Figure 3:**
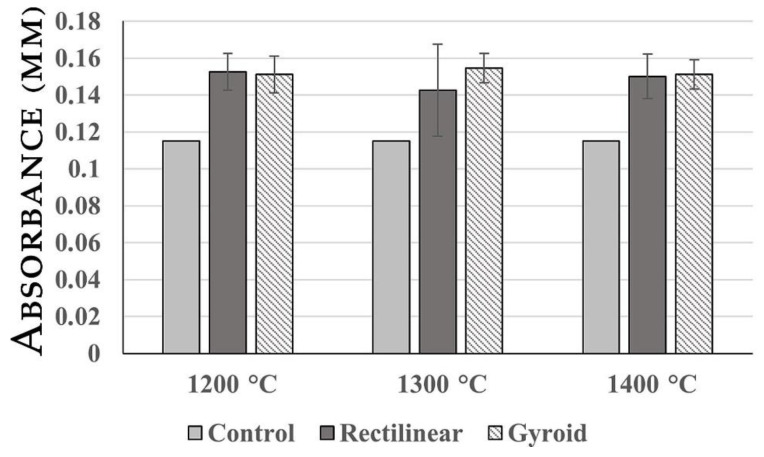
Results f the LDH release assay. LDH accumulation changes in the case of all analyzed scaffolds were insignificant, suggesting good biocompatibility.

LDH release assay was used to estimate material cytotoxicity by releasing LDH as a substitute marker for membrane disruption in human cells. The results of the LDH release assay presented in Figure 3 demonstrated that all analyzed materials showed a slight increase in LDH leakage of ASCs into the culture medium.

### 2.3. Characteristics of Used Material

**Figure 4:**
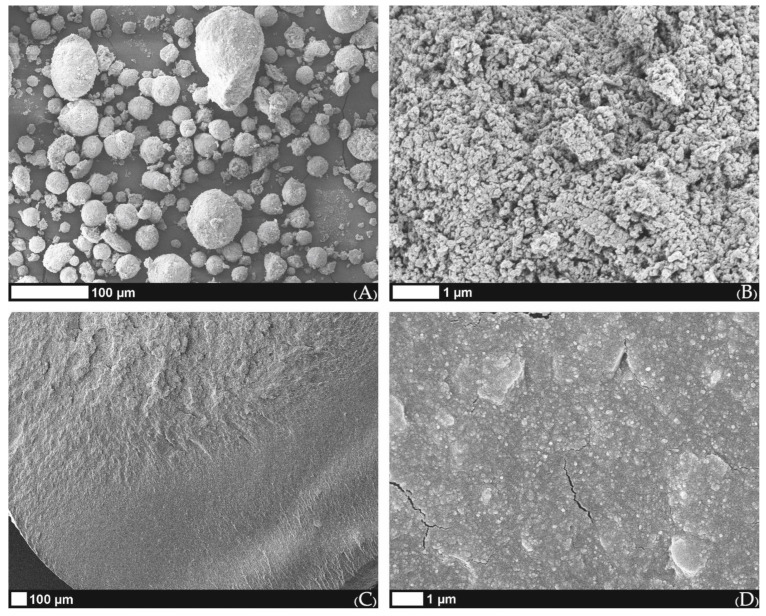
SEM images of (**A**) spherical aggregates of used CDHA; (**B**) enlarged surface image of the CDHA aggregate; (**C**) filament fracture surface; (**D**) enlarged fracture surface image with several fissures produced during filament fracturing under cryogenic conditions.

The morphology of calcium-deficient hydroxyApatite (CDHA) particles observed under SEM revealed almost spherical aggregates (Figure 4a) that were composed of primary HA crystals around 100 nm in size when the top surface of the aggregate was observed (Figure 4b). Perpendicular filament fracture areas made under cryogenic conditions showed homogeneous surface characteristics with well-distributed CDHA in a used polymeric binder (Figure 4c). Indeed, the magnified fracture surface (15,000×) (Figure 4d) shows that good distribution of primary HA crystals was achieved from CDHA aggregates during filament preparation, while cryogenic filament fracturing also generated some fissures longer than 1 µm (Figure 4d).

**Figure 5:**
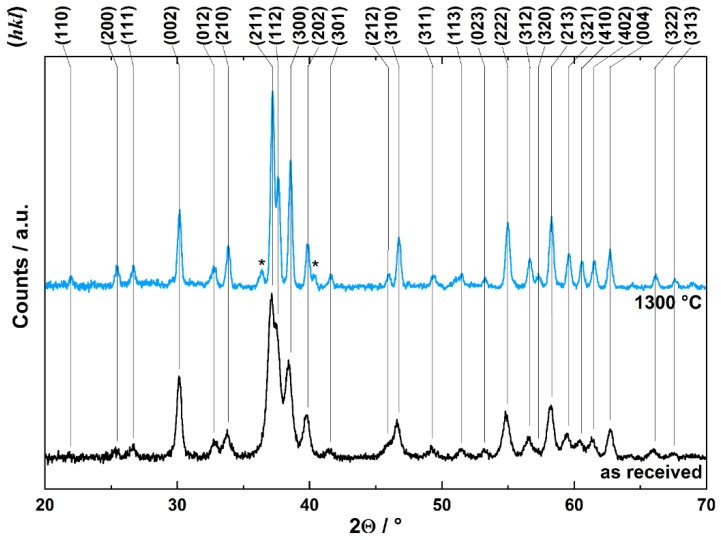
X-ray diffraction patterns of as received CDHA (**bottom**) and after the material sintering at 1300 °C (**top**) with respective (hkl) indexes. Asterisks describe two diffractions of lesser intensity observed at 36.4 and 40.3° 2Θ.

For biomedical applications, the comprehensive characteristics of investigated CDHA powder are important indicators of specific physical–chemical characteristics of the materials used. The XRPD pattern of CDHA revealed relatively broad diffraction peaks for low crystalline HA structure with the corresponding (hkl) indexes (Figure 5). The evaluation in Match!^©^ software (Crystal Impact GbR, Bonn, Germany) confirmed the hydroxyapatite structure according to the Crystallography Open Database entrance 96-900-1234 (COD-Inorg 2021.06.14). The effect of heating on CDHA crystal structure is reflected by the XRPD pattern shown in Figure 5, and the selected temperature was 1300 °C. The heating induced the narrowing of diffraction peaks, indicating an improvement in HA crystallinity. At the same time, two diffractions of lesser intensity are observed at 36.4 and 40.3° 2Θ (denoted by stars in Figure 5). These two diffractions correspond to the new phase developed by heating and correspond to the most intense diffraction peaks of β-TCP produced by the dehydroxylation reaction of HA [42]. Hence, the sintering of CDHA to the desired temperature of 1300 °C forms biphasic calcium phosphate biomaterial with changed bioactivity [43].

**Figure 6:**
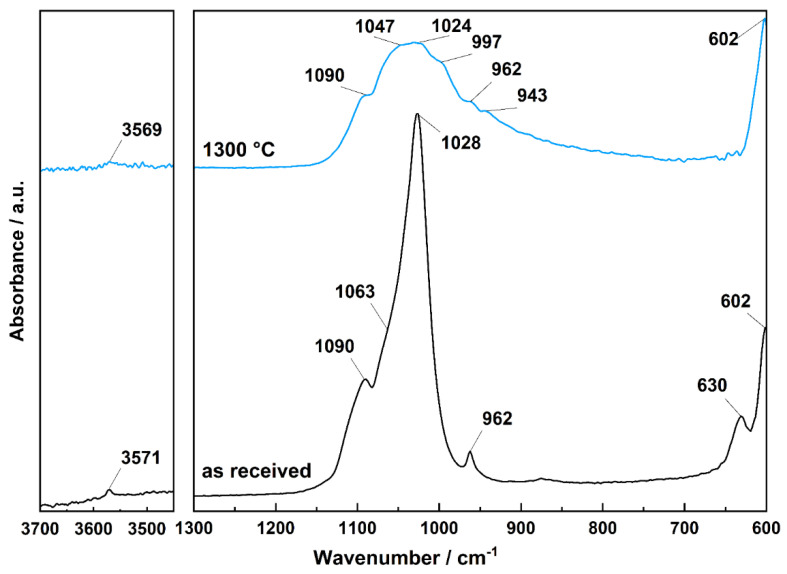
ATR-FTIR spectra of as received CDHA (**bottom**) and corresponding spectra after the material sintering at 1300 °C (**top**).

The characteristics of CDHA were also investigated by ATR-FTIR spectroscopy. The spectrum of original CDHA as received from the supplier is shown in Figure 6; band identification was proven by structure isotopic substitution by Fowler (1974) [44], as follows: the band observed at 3571 cm^−1^ corresponds to the OH stretching mode, while the librational mode of OH groups is observed at 630 cm^−1^; the bands observed at 1090 cm^−1^ and 1028 cm^−1^ including shoulder around 1063 cm^−1^ correspond to triply degenerated ν_3_ antisymmetric PO_4_^3−^ stretching mode, the band observed at 962 cm^−1^ represent ν_1_ nondegenerate PO_4_^3−^ symmetric stretching vibration, the band at 601 cm^−1^ is the component of triply degenerate ν_4_ O–P–O bending mode vibration. The lower frequency bending modes are not shown due to the absorption edge of the ZnSe crystal used in the ATR module. After CDHA sintering at 1300 °C, the band intensities corresponding to stretching and librational modes of structural OH groups strongly decreased, as expected, due to the structural dehydroxylation of CDHA (Figure 6). The band intensities of PO_4_^3−^ anions also decreased, and the triply degenerated ν_3_ antisymmetric PO_4_^3−^ stretching bands are resolved and detected at 1090 cm–1, 1047 cm^−1^, and 1024 cm^−1^, and newly developed sidebands at 997 and 943 cm^−1^ of the band observed at 962 cm^−1^ may represent ν_1_ degenerate PO_4_^3−^ symmetric stretching vibrations.

### 2.4. Surface Morphology of Scaffolds Sintered at Elevated Temperatures

**Figure 7:**
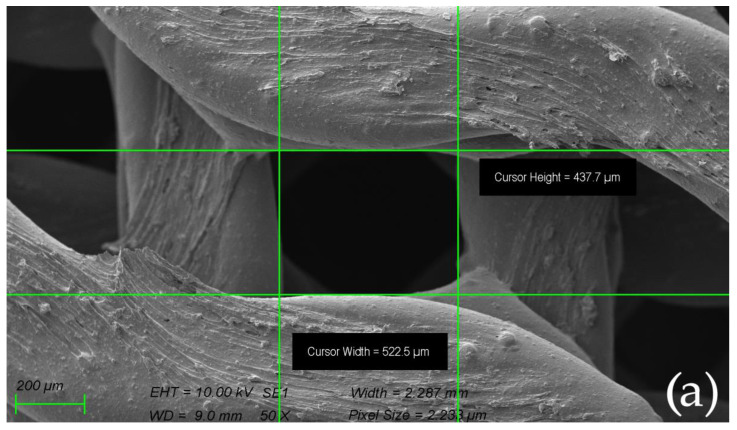

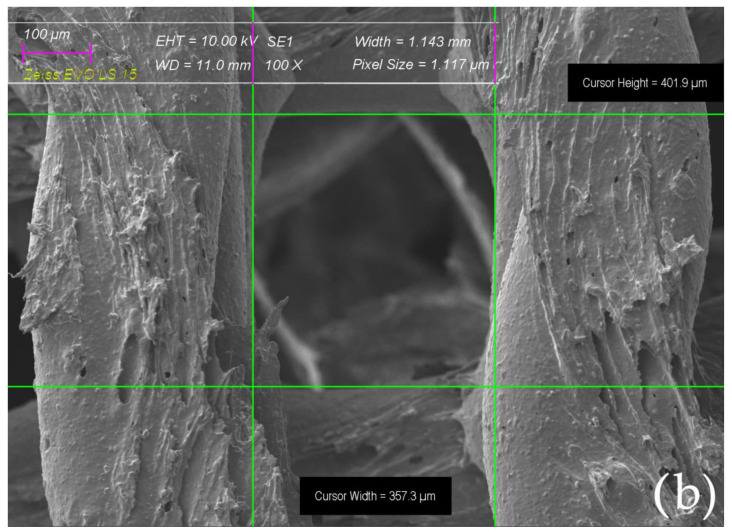
The morphology of the scaffolds observed by SEM before biocolonization with MSCs: (**a**) gyroid scaffold measured before sintering; (**b**) gyroid scaffold measured after sintering in 1400 °C.

The morphology of the scaffolds before and after sintering observed by SEM is shown in Figure 7. The scaffolds printed from composite filament sintered at elevated temperatures of 1200, 1300, and 1400 °C revealed linear shrinkage when compared to the green body, corresponding to values ranging from ~30 to 33%.

### 2.5. Resulting Scaffolds after Biocolonization with MSC

**Figure 8:**
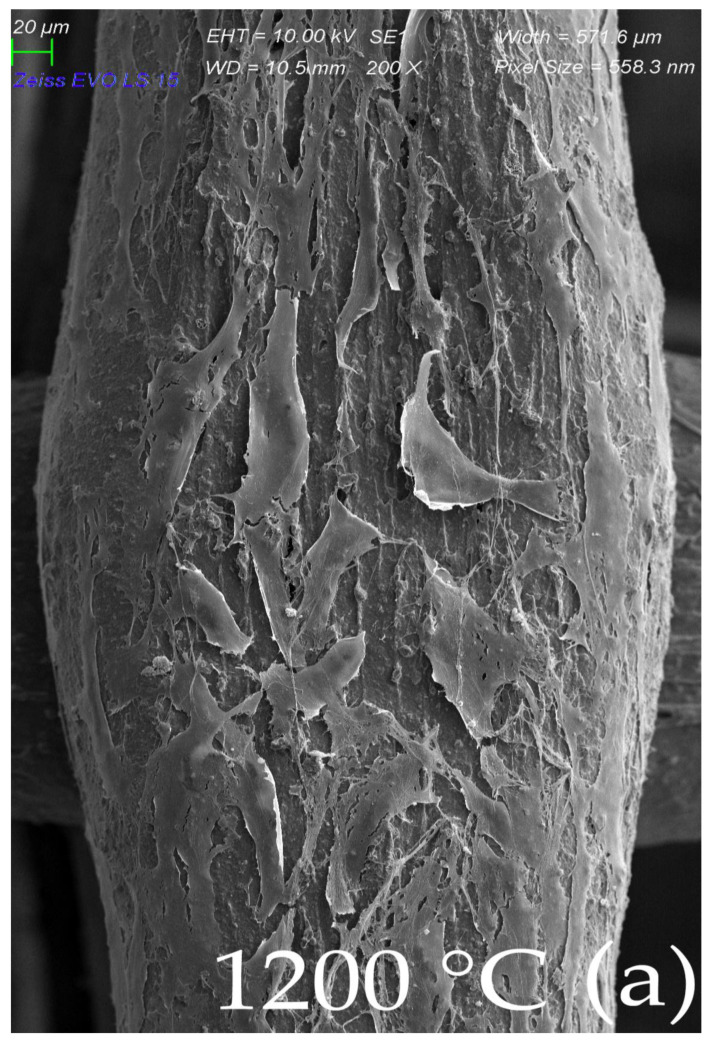

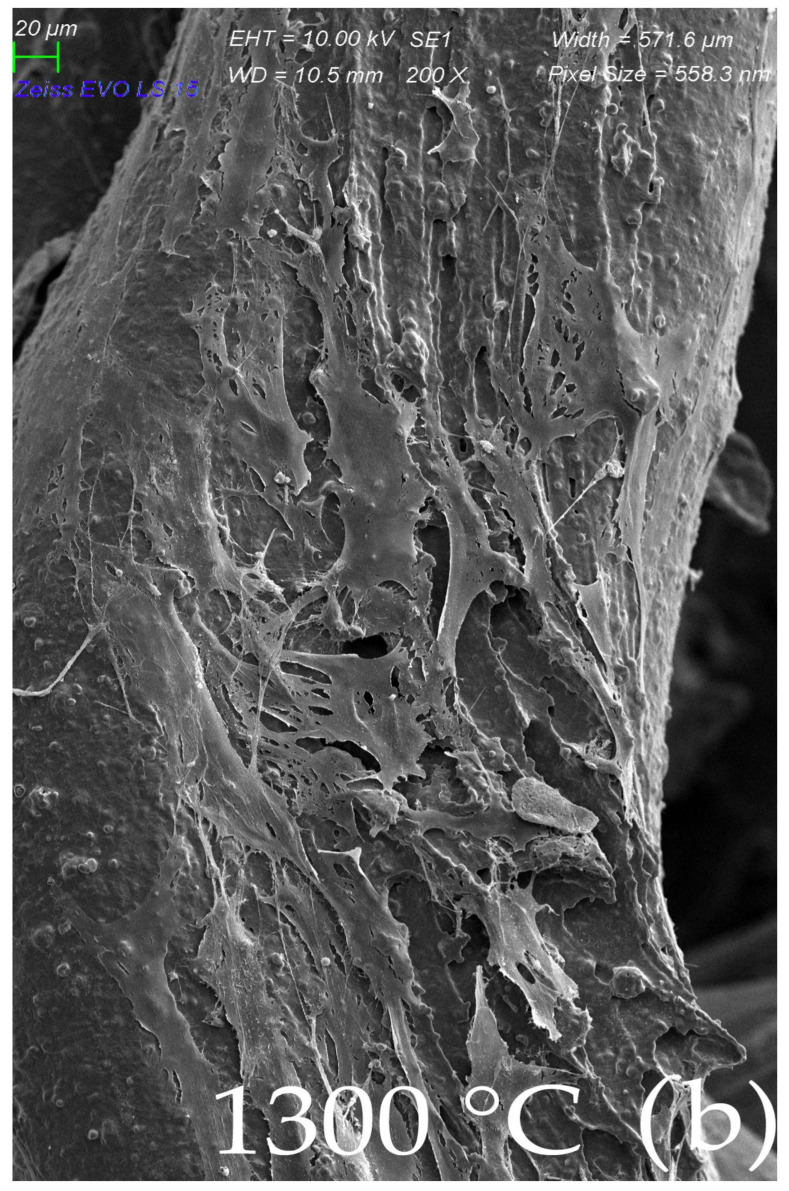

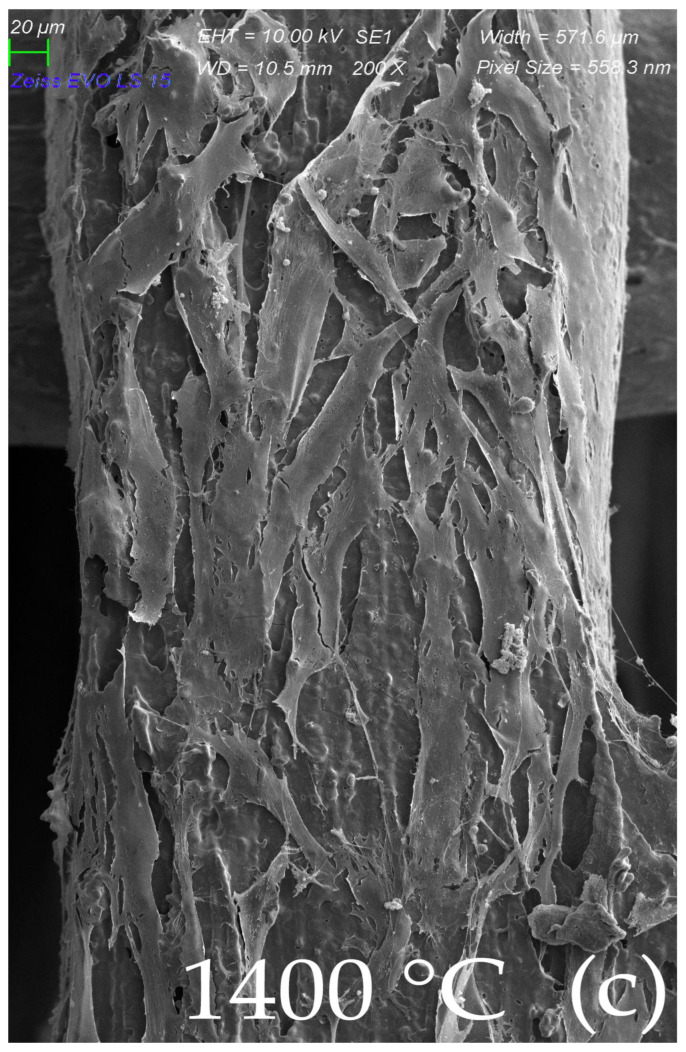
Morphological SEM evaluation of biocolonization evaluated gyroid scaffolds sintered in three different temperatures (1200, 1300, and 1400 °C): (**a**) Surface of MSCs culture, cultivated on scaffold sintered at 1200 °C. SEM, Mag ×200. Flat cells are sparsely distributed throughout a scaffold line surface. Long elongated projections connect to the line surface and to each other; (**b**) Surface of MSCs culture, cultivated on scaffold sintered at 1300 °C. SEM, Mag ×200. Similar patterns of cell distribution projections overlap more frequently; (**c**) Surface of MSCs culture, cultivated on scaffold sintered at 1400 °C. SEM, Mag ×200. A monolayer of flat cells with intensive cell overlapping and few of them with vesicles probably due to most beneficial conditions.

There was no significant difference between the scaffolds sintered at elevated temperatures of 1200, 1300, and 1400 °C in terms of the architecture of the scaffold. The morphology of the MSC-populated scaffolds after sintering can be seen in Figure 8a–c (SEM). The surface of MSCs, cultivated on the scaffold sintered at 1200 °C presented flat cells sparsely distributed over the surface of the scaffold, which was well observed by SEM. Long, elongated projections were connected to the surface and to each other (Figure 8a). Cells cultivated on a scaffold sintered at 1300 °C showed similar patterns of cell distribution, with more frequent overlapping projections (Figure 8b). The surface of the MSC culture cultivated on a scaffold sintered at 1400 °C showed a monolayer of flat cells with intense cell overlap and only a few of them with vesicles, probably due to the favorable conditions (Figure 8c).

**Figure 9:**
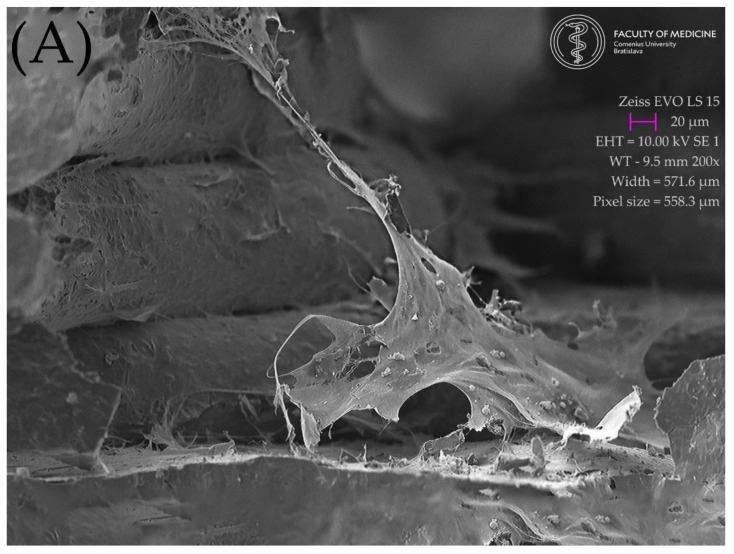

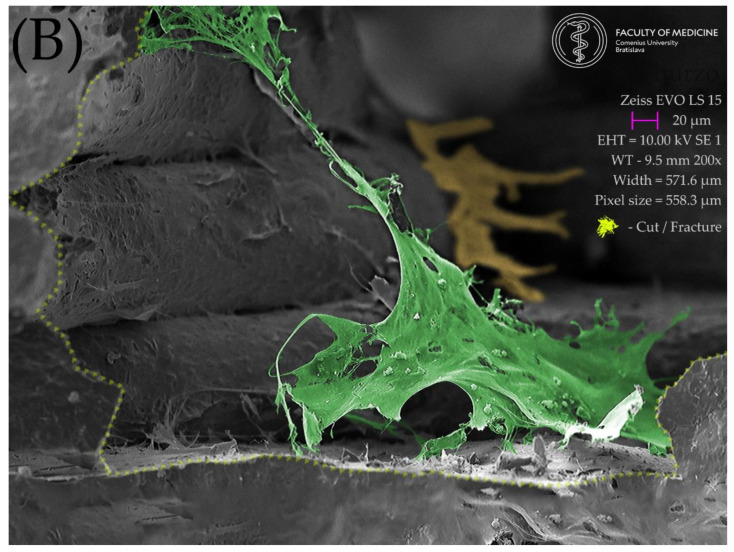
Lateral view of the interior of the scaffold floor, created with an ultra-sharp razor blade. A large flat cell in the foreground shows exceptionally long cell projections firmly attached to the lateral surfaces of the scaffold. The openings between the scaffold lines appear to be a friendly environment for cell growth: (**A**) original SEM image; (**B**) enhanced image showing the scaffold section and the green-colored cell attached to the scaffold from bottom to the top, with a yellow-colored cell in the background climbing up in the posterior corner of the scaffold.

A monolayer of flat elongated or star-shaped cells covered the surface of the scaffold. Cell cultures were found on the scaffold material at the top, the bottom, and even on its lateral sides. Numerous projections protruded from the cells. Long projections helped the cells to adhere to the scaffold surface and to interact with each other, as well. Several short projections overlapped the body of the neighboring cell. Some cells showed a smooth surface, and some of them reflected an outgrowth of cell projections, with the appearance of a rough surface with vesicles. Preservation of the cell population up to the 14th day after the seeding of the cell culture was also observed with scaffolds fired at a temperature of 1200 °C, but the population with the highest density of cells was observed for the scaffold fired at 1400 °C. Figure 9. Shows a view of the interior of scaffold cut by an ultra-sharp razor blade. A large flat cell in the foreground (colorized green Figure 9b) shows exceptionally long cell projections firmly attached to the lateral surfaces of the scaffold. The openings between the scaffold lines appear to be a friendly environment for cell growth: The original SEM image is show in Figure 9a and the enhanced image in Figure 9b. It shows the green-colored cell attached to the scaffold from bottom to the top and also a yellow-colored cell in the background climbing up in the posterior corner of the scaffold.

## 3. Discussion

The results of this research can be interpreted as a promising signal to future perspectives of regenerative dentistry addressing the needs of bone defect therapy. The findings showed that the material could be utilized in FDM manufacturing scaffolds of desired porosity with reliable geometry precision. Implications of this conclusion are important for future 3D personalization of the colonized object intended for bone defects of specific shape and size.

The findings regarding cytotoxicity of the material showed in the LDH release assay and MTT assay that all analyzed materials were non-toxic, which implicates the suitability of this material for biocolonization and, thus, a possible path for desired cellular personalization.

Reflecting on why the MTT and LDL assays were different at 1200 °C, we can assume that at a temperature of 1200 °C the remnants of the toxic substances might have been preserved, resulting in the inhibition of cell proliferation, but, on the other hand, having no effect on membrane fragmentation, which was not demonstrated by the LDH release assay.

Implications of these findings in the broadest context possible suggest a feasible path in future clinical applications of this material and methods. It may also be highlighted that this paper introduces the interdisciplinary cooperation of material scientists, tissue engineering experts from the fields of biology and medicine, and dentists focused on regenerative dentistry. This cooperation towards the utilization of this novel HA material with 3D FDM printing and MSC colonization presents a prospective setup for the repair of critical size in alveolar bone defects, which is still an unmet clinical need.

In similar research published by González-Henríquez et al. (2022), an attempt was made to create a cellular scaffold with an intricate and complex network of interconnected pores and microchannels using salt leaching and additive manufacturing (3D printing) methods that mimic the hierarchical internal structure of bone. A biocompatible hydrogel film (based on polyethylene glycol) was used to cover the top of various polymeric scaffolds. The pores had a mean size of 26.4 ± 9.9 μm, resulting in a total scaffold porosity of ~42% (including pores and microchannels), which is similar to the porosity of scaffolds used in this study (50% porosity achieved by larger pores 350–400 μm) [45]. Limitation of scaffold thickness for clinical applications persists due to the anticipated lack of vascularization in the in vivo environment.

Another recent study published in the *Journal of Functional Biomaterials* by Mocanu et al. (2022) presented the intersection of bone tissue reconstruction and additive manufacturing fields through the introduction of high-performance bone-like scaffold manufacturing. However, the strategy proposed in this paper was directed toward the use of bovine-bone-derived hydroxyapatite for surface properties enhancement and mechanical features reinforcement of the polylactic acid (PLA) matrix for composite filament extrusion. Mocanu et al. have also used SME for the analysis of melt mixtures of HA + PLA and found uniform and homogenous dispersion of HA particles and adequate adhesion at the ceramic/polymer interface without outline pores [46].

On the contrary, in our study, we utilized CDHA composed of primary HA crystals around 100 nm in size prior to sintering. These were used in the preparation of the composite filament with a final 50 wt.% of CDHA and 50 wt.% of a thermoplastic binder, including polyvinyl alcohol and plasticizer [47]. The polyvinyl alcohol was used only as a thermoplastic binder and was eliminated during the thermal debinding process. A possible explanation of why the material prepared at 1200 °C had an inhibitory effect on cell proliferation is the hypothesis that some toxic remnants of the PVA produced by thermal debinding were not completely removed during the sintering process, albeit this is not yet an approved hypothesis.

In the clinical comparison of synthetic scaffolds and autografts in animals, a recent study by Rahyussalim et al. (2022) presented the in vivo use of 3D PLA scaffolds with HA/alginate composite injection and MSC as laminoplasty spacers in rabbits. This study investigated the in vivo biocompatibility and tissue scaffold integration of a PLA scaffold with the addition of alginate/HA and MSC injections. This shows that the synthetic scaffolds we used had a similar tissue response and may have a tissue integration profile as the autografts [48]. The results presented in this paper shall encourage further scientific research and, in perspective, translational studies in animals and later in humans so that this biocompatible scaffold can be developed to fill bone defects [22,49,50].

To address a final intriguing observation was the search for an explanation for the sudden bluish coloration of originally white scaffolds after the firing process. A possible explanation of this phenomenon is provided by previous research. As the scaffold contains traces of manganese, a possible explanation for the blueish dye of the fired scaffolds is its oxidation, which occurs during the sintering process in an oxygen-containing atmosphere. These phenomena have also been observed in a paleontological study searching for answers to bluish bones found. For example, results of the investigation of paleontological blue and gray bone fragments of small vertebrates coming from stratigraphic layer 770 at San Josecito Cave (Nuevo Leon, Mexico, dating between 28,000 and 19,000 years BP). Furthermore, prior research has shown that Mn^5+^ in tetrahedral coordination could be responsible for the turquoise blue color in mastodon ivory, some tens of million years old, that was affected by heat. Manganese is present in the anionic form of (MnO_4_)^3−^ and partially substitutes for (PO_4_)^3−^ in the hydroxyapatite matrix. Cations of Mn^5+^ in a tetrahedral environment of four O^2−^ ions in the apatite structure are found in bluish bones at San Josecito Cave, the same color origin as in the blue mastodon ivory. The formation of Mn^5+^ is likely induced by heat treatment of the bones under oxidizing conditions [51].

**Figure 10:**
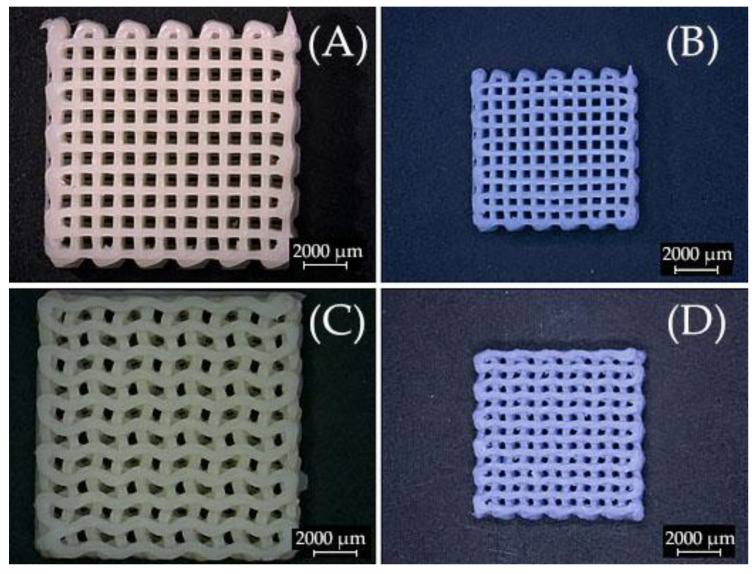
Optical microscopy images of 3D printed scaffolds (**A**) rectilinear as printed; and (**B**) sintered at 1300 °C; (**C**) gyroid as printed; and (**D**) sintered at 1300 °C.

Figure 10 shows examples of optical microscopy of 3D printed scaffolds rectilinear/gyroid as printed (Figure 10a,c); and sintered at 1300°C with a touch of blue hue (Figure 10b,d).

## 4. Materials and Methods

### 4.1. Materials

Commercially available calcium deficient (CDHA) powder (Ca/P ≅ 1.54, Brenntag, Netherlands B.V.) with spherical particle aggregates having diameters ranging from ~1 up 200 µm with d_50_ ≤ 35 µm determined by light scattering measurements using Malvern Instruments Ltd. Mastersizer 3000 was used. The hydroxyapatite (HA) was applied as received for the preparation of composite filament from 50 wt.% of CDHA and 50 wt.% of thermoplastic polymer, polyvinylalcohol (PVA, Kuraray-POVAL^TM^, Frankfurt am Main, Germany) including plasticizer [47]. The filament with a diameter of 1.75 ± 0.05 mm was extruded using above mentioned pre-mixed components in a double screw extruder, and the desired filling grade of the inorganic component after extrusion was also approved by the gravimetry after composite firing at 1300 °C to be 47.0 wt.%. The water loss in CDHA determined by thermogravimetry showed gradual water loss from room temperature (20 °C) until the expected dehydroxylation temperature (800 °C) with a mass change of 3.78%. This value is already above the theoretical amount of water released from stoichiometric hydroxyapatite (STHA Ca/P ≅ 1.664) reported by Markovic et al. 2004 [52]. The most water loss in this temperature range is associated with the release of adsorbed surface water. Water formed by CDHA structure dehydroxylation resulting in the formation of β-tricalcium phosphate (β-TCP) during the highest temperature treatments from the investigation range used (20–850 °C), reported by Markovic et al. (2004), was on the level of 6.5% of total water amount released. The CDHA and thermoplastic binder in a mass ratio of 1:1 were homogenized using a twin screw extruder (Compuplast^®^, Zlín, Czech Republic). The prepared filament was cut into pellets, and these were reused for filament production in the same twin-screw extruder. The nominal filament diameter of 1.75 mm ± 0.05 mm required for commercial FFF printers was extruded. The chemical composition of HA with Ca/P = 1.53, according to the certificate of analysis, was: 52.2 ± 0.7 wt.% CaO; 43 ± 5.0 wt.% P_2_O_5_; 8.0 ± 0.1 wt.% H_2_O; ~50 ppm F; ~650 ppm metals.

### 4.2. Methods

The X-ray powder diffraction (XRPD) and Fourier transform infrared spectroscopy (FTIR) for better assignment of material properties and a clear comparison with past and future investigations was used. The CDHA structure changes induced by material sintering at elevated temperatures were investigated by XRPD using the Brag-Bretano geometry of a Stoe Thetha–Thetha goniometer equipped with a linear position-sensitive detector. The cobalt lamp radiation CoKα (1) with a wavelength of 0.1788965 nm and measuring range of 20–70 2θ° with a 0.2 step and integration time of 10 s was used. The XRPD patterns were evaluated using the software Match!^©^ ver. 3.12 and Crystallography Open Database (COD-Inorg 2021.06.14) entrance 96-900-1234.

The spectral features of CDHA in the mid-infrared region were revealed by infrared spectroscopy measured by ATR module PIKE MIRacle™ with ZnSe crystal using an FTIR spectrometer Nicolet 6700 and DTGS detector. The spectra were measured in the range of 4000–600 cm^−1^ (due to the ZnSe absorption edge at ~600 cm^−1^), averaging 64 spectral scans with a resolution of 4 cm^−1^. The background signal was measured prior to each measurement, and spectra were evaluated by Omnic^®^ (Thermo Fisher Scientific Inc., Waltham, MA, USA) software ver. 7.1 (Figure 6). The water loss from HA powder was followed by thermogravimetric analysis using TG/DTA 6300 EXSTAR SII Hitachi (Hitachi High-Tech Science Corporation, Tokio, Japan). The scaffold surface features and changes in the microstructure of the sintered material were observed by scanning electron microscopy (SEM) using a JEOL 7500 F (JEOL Ltd., Tokyo, Japan) with an accelerating voltage of 15 kV. The powder or part of the scaffold was stuck on conductive tape, and the surface of non-conductive ceramic was covered by gold in a vacuum evaporator. The tested scaffolds were printed on a Leapfrog^TM^ Creatr 3D printer controlled from a PC by the Repetier-Host software version 2.2.2 (Hot-World GmbH & Co. KG, Willich, Germany). Scaffolds with rectilinear or gyroid architecture of individual layers were deposited on the printing platform with the size of the composite green body after printing 12.5 × 12.5 × 2.6 mm^3^. A simple STL model of the test plate was created in the free available Tinkercad program, while the printing conditions and slicing were set in Repetier-Host using the slicer software Slic3r version 1.3.1. program with following parameters: printing speed of 20 mm∙s^−1^; Filament extrusion multiplier of 1.00×; infill—rectangular/gyroid; filling density—42%, 48%, 52%/41%, 46%, 50% (rectilinear/gyroid); layer height—0.2 mm; extruder temperature—220 °C; pad temperature—none; nozzle diameter—0.4 mm. The scaffolds were thermally debinded in a static air atmosphere using a programable oven Classic^TM^ (Czech Republic). The debinding program was optimized according to the thermal decomposition of composite filament observed under its thermogravimetric behavior observed with a heating rate of 10 °C∙min^−1^. Hence, the slowest thermal heating rates equal to 0.1 °C∙min^−1^ required during debinding in the oven were at temperatures ~250 and ~400 °C [53]. Optical microscope images of scaffolds were collected prior to and after sintering using an optical microscope Zeiss Stemi 508. This was equipped with an objective set enabling enlargements ranging from 0.63 to 5× and coupled with an Axiocam 105 (Carl Zeiss AG, Jena, Germany).

#### 4.2.1. Direct Contact Cytotoxicity Assay

Cell proliferation and morphological changes were studied using an inverted light microscope (Zeiss Axiovert 100, Carl Zeiss, Jena, Germany).

#### 4.2.2. MTT Assay

The 3-(4,5-dimethylthiazol-2-yl)-2,5-diphenyltetrazolium bromide (MTT) assay (The CellTiter 96^®^ AQueous One Solution Cell Proliferation Assay, Promega, Madison, WI, USA) was used to analyze cell viability, for detecting the cytotoxicity and effect of material on cell growth. Sterile scaffolds were placed into a 24-well plate and rinsed with complete culture medium DMEM Low glucose supplemented with 10% FBS, 100 µg/mL streptomycin, and 100 U/mL penicillin (Sigma-Aldrich, St. Louis, MO, USA). Afterward, adipose tissue-derived stem cells with a density of 5 × 10^4^ cells per well were seeded on the scaffolds, followed by incubating at 37 °C for 48 h. At the end of the incubation, 10 µL of MTT solution was added, followed by 4 h incubation. The supernatant was aspired and transferred into a new plate, and absorbance was recorded at 490 nm using a plate reader BioTek EL800 (BioTek, Winooski, VT, USA). All experiments were performed in triplicate.

#### 4.2.3. LDH Release Assay

Cytotoxicity induced by scaffolds was evaluated by lactate dehydrogenase (LDH) release assay (Sigma-Aldrich, St. Louis, MO, USA). LDH assay mixture was prepared by mixing equal volumes of LDH assay substrate solution, LDH assay dye solution, and 1× LDH assay co-factor preparation. An aliquot of the medium was aspirated, and a lactate dehydrogenase assay mixture was added to each sample. The plate was covered with an opaque material to protect against light and incubated at room temperature for 20–30 min. Absorbance was measured spectrophotometrically at the wavelength of 490 and 630 nm using a plate reader BioTek EL 800. All experiments were performed in triplicate.

#### 4.2.4. Methods of Sample Preparation for Scanning Electron Microscopy

The cell cultures adhered on scaffolds in culture medium were gently washed with 3% glutaraldehyde buffered solution fixative for 30 min at room temperature. Afterwards, samples were rinsed three times in phosphate buffer solution and postfixed in osmium tetroxide solution for 1 h at 4 °C temperature. After the rinse in demineralized water, samples were dehydrated through a graded ethanol series to 100% ethanol, followed by critical point drying of liquid CO_2_. Finally, they were mounted on aluminum specimen stubs with carbon adhesive tapes, sputter coated with a 7 nm layer of gold/palladium, and examined with a scanning electron microscope ZEISS type EVO LS 15.

#### 4.2.5. Statistical Analysis

All quantitative results were obtained from at least triplicate samples. Data are expressed as the mean ± standard deviation. Statistical analysis between groups was conducted using single-factor analysis of variance (ANOVA). A value of *p* < 0.05 was considered to be statistically significant.

## 5. Conclusions

This proof-of-concept study presents a feasible fabrication of CDHA/PVA composite filament, FDM 3D printing of CDHA scaffold, thermal post-processing and sintering of the scaffolds, and subsequent colonization with MSC. In vitro characterization of the material with MTT and LDH assays showed that the resulting CDHA-based scaffold was non-toxic, biocompatible, and exhibited good cell adherence, which was morphologically evaluated by SEM.

Gyroid or rectilinear geometric arrangement of the pores did not significantly affect cell colonization and adhesion, but higher temperatures during the sintering of the scaffold were better for cell colonization. Scaffold fired at the lowest temperature of 1200 °C even showed an inhibitory effect on cell proliferation.

In conclusion, the novel 3D-printed hydroxyapatite scaffolds have great potential for use in the tissue engineering of bone. However, further in vitro and in vivo studies on their osteoinductive potential and biological safety need to be performed to support translation into clinical applications.

## Data Availability

Not applicable.
